# Interactive effect of soil moisture content and phosphorus fertilizer form on chickpea growth, photosynthesis, and nutrient uptake

**DOI:** 10.1038/s41598-022-10703-0

**Published:** 2022-04-23

**Authors:** Mohamed Chtouki, Fatima Laaziz, Rachida Naciri, Sarah Garré, Frederic Nguyen, Abdallah Oukarroum

**Affiliations:** 1 Plant Stress Physiology Laboratory, Mohammed VI Polytechnic University (UM6P) – AgoBioSciences, Lot-660 Hay Moulay, Rachid, 43150 Benguerir, Morocco; 2 University of Liege - Gembloux Agro-Bio Tech Faculty, 5030 Gembloux, Belgium; 3 School of Engineering, University of Liege - UR UEE, 4000 Liege, Belgium; 4 High Throughput Multidisciplinary Research Laboratory, Mohammed VI Polytechnic University, 43150 Benguerir, Morocco

**Keywords:** Photosynthesis, Plant development, Plant physiology, Plant stress responses

## Abstract

Water shortage and soil nutrient depletion are considered the main factors limiting crops productivity in the Mediterranean region characterized by longer and frequent drought episodes. In this study, we investigated the interactive effects of P fertilizer form and soil moisture conditions on chickpea photosynthetic activity, water and nutrient uptake, and their consequent effects on biomass accumulation and nutrient use efficiency. Two P fertilizer formulas based on orthophosphates (Ortho-P) and polyphosphates (Poly-P) were evaluated under three irrigation regimes (I1: 75% of field capacity, I2: 50% FC and I3: 25% FC), simulating three probable scenarios of soil water content in the Mediterranean climate (adequate water supply, medium, and severe drought stress), and compared to an unfertilized treatment. The experiment was conducted in a spilt-plot design under a drip fertigation system. The results showed significant changes in chickpea phenotypic and physiological traits in response to different P and water supply regimes. Compared with the unfertilized treatment, the stomata density and conductance, chlorophyll content, photosynthesis efficiency, biomass accumulation, and plant nutrient uptake were significantly improved under P drip fertigation. The obtained results suggested that the P fertilizer form and irrigation regime providing chickpea plants with enough P and water, at the early growth stage, increased the stomatal density and conductance, which significantly improved the photosynthetic performance index (PI_ABS_) and P use efficiency (PUE), and consequently biomass accumulation and nutrient uptake. The significant correlations established between leaf stomatal density, PI_ABS_, and PUE supported the above hypothesis. We concluded that the Poly-P fertilizers applied in well-watered conditions (I1) performed the best in terms of chickpea growth improvement, nutrient uptake and use efficiency. However, their effectiveness was greatly reduced under water stress conditions, unlike the Ortho-P form which kept stable positive effects on the studied parameters.

## Introduction

Drought and soil nutrient depletion are considered the main factors influencing crop productivity in the Mediterranean region^1,2^. The impact of climate change on agriculture in the arid and semi-arid regions is increasingly felt, through the negative impacts of the decrease in precipitation, the high temperatures and radiation, and the longer and frequent drought on crop yield and quality^3^. Under these challenging pedoclimatic conditions, plants showed a series of morphological, physiological, and biochemical modifications to adapt to different types of abiotic stress and improve their capacity to absorb and use water and mineral resources efficiently^4–6^. It is widely documented that plants exposed to drought and nutrient deficiency reduce their photosynthetic activity and gas exchanges with the environment, by reducing the leaf area, closing their stomata, and increasing assimilates allocation to the root system^4,6–9^.

Phosphorus (P) is the second macronutrient limiting crops productivity in alkaline soils, mainly under arid and semiarid areas, due to its rapid complexation and precipitation with soil cations^10–12^. Although the application of P fertilizers has significantly improved crop yields through the enhancement of several biophysiological processes in plants, P is still one of the main nutrients showing low use efficiency^13,14^. In some cases, excessive amounts of P fertilizer are used to ensure high crops yield, which can consequently lead to abusive use of this non-renewable resource and damage the ecosystem^15^. P use efficiency is mainly dependent on P mobility and availability in the soil^16,17^. The capacity of plants to take up P is strongly related to many soil characteristics and parameters as well as to the fertilizer properties such as pH, fertilizer form and solubility and its interaction with the soil moisture and compounds^18–20^. For example, the application of orthophosphate (Ortho-P) fertilizers under high alkaline soil conditions resulted in high interaction between P ions and other bivalent cations, which can lead to the formation of some insoluble P forms, and consequently reduce P uptake and recovery by plants^21^.

To deal with this major challenge, several strategies have emerged to improve P use efficiency, including the development of new fertilizer formulas and the implementation of high-frequent drip fertigation practices, as well as P foliar application and the use of plant growth-promoting rhizobacteria (PGPR) and biostimulant^14,22,23^. With this regard, increasing attention was paid to polyphosphates (Poly-P) fertilizers due to their high solubility and slow-release properties^23–26^. The Poly-P, which are defined as a condensed form of P, are usually less reactive in soil compared to the Ortho-P forms and present some interesting properties such as their capacity to chelate some micronutrients, especially under alkaline soil conditions^27^. Despite, these beneficial effects of Poly-P on nutrient availability and crops yield, their effectiveness is drastically influenced by soil properties and agricultural practices such as soil moisture and water irrigation amount and scheduling^25,28^.

Chickpea is a legume crop cultivated in the Mediterranean basin for its high nutritional value and its capacity to grow in harsh environments^29–31^. However, its response to P and water supply is greatly dependent on P fertilizer forms and water application strategies^29,32,33^. Even though drip fertigation is a highly efficient technic to maximize chickpea production, some issues related to the choice of fertilizers forms, rates, application timing and their interaction with soil moisture content are still of high interest for farmers, agronomists, and plant physiologists. It should be noted that most studies dealing with the interaction between water and P have mainly focused on P availability in soils and its direct effect on crops yields^19,34,35^. More attention can be drawn to the effect of P fertilizer forms, in different irrigation scenarios, on the photosynthetic apparatus, leaf stomatal activity, nutrient uptake and allocation, and their consequent effects on biomass accumulation and crops yield.

The objective of this study was to investigate the interactive effect of P fertilizer form and soil moisture content on the main biophysiological process influencing chickpea growth. Three P fertigation regimes (zero P supply, Ortho-P, and Poly-P) were evaluated under three irrigation scenarios simulating three soil water content levels (75% of field capacity; 50% FC, and 25% FC). The drought stress regimes were applied on 1-month-old plants and maintained for 32 days. Emphasis was placed on how Poly-P fertilizers can contribute to improving P use efficiency and crops yield under Mediterranean conditions characterized by frequent drought events.

## Materials and methods

### Experimental conditions, plant material and treatments

The experiment was conducted under greenhouse conditions at the experimental farm of the Mohammed VI Polytechnic University, in Benguerir central Morocco (32° 13′ 11.5′′ N 7° 53′ 29.9′′ W) which is a semi-arid region, with a mean monthly rainfall of 13.18 mm, of which approximately 7.75 mm fall in spring mm. The experiment was conducted in the 2021 spring chickpea growing season, with an experimental design composed of 54 pots (cylindrical pot: 10 L volume, 24 cm diameter, 21 cm height) containing 10 kg of sandy–clay–loam soil per pot. The studied soil is characterized by 0.9 g kg^−1^ of total nitrogen, 26 mg kg^−1^ of Olsen-P, 246 mg kg^−1^ of exchangeable K_2_O, 10.6 g kg^−1^ of CaO, 427 mg kg^−1^ of MgO, pH 8.4, 1.4% of organic matter, and a bulk density of 1.2 g cm^−3^. Further details regarding soil physicochemical properties are reported in Chtouki et al.^25^. The chickpea was planted at a density of 9 seeds per pot on February 11th, 2021, then thinned 3 days after plant emergence to ensure a homogeneous population density of 3 plants per pot, equivalent to 30 plants m^−2^. Chickpea plants were harvested on April 14th. Chickpea seeds (*Cicer arietinum* L.) were obtained from the Moroccan National Seed Marketing Company (SONACOS). This variety used for the experiment was “Kabuli Moubarak”, which is widely planted in Morocco.

During the first 30 days from sowing, chickpea plants were grown under adequate soil moisture conditions (75% of soil FC). After that, three different irrigation regimes: irrigation at 75% of field capacity (FC) (I1), irrigation at 50% of FC (I2), and irrigation at 25% of FC (I3) were applied (until the harvest), based on regular measurements of soil volumetric water content (VWC). The soil VWC was measured by the soil moisture meter WET-2 sensor (Delta-T devices Ltd, USA) that reads and stores measurements. The amount of required water for each irrigation regime was calculated every 2 days to maintain the soil at the desired field capacity level. In each irrigation regime, two inorganic P fertilizers, orthophosphate (Ortho-P) or polyphosphate (Poly-P) were applied at rate of 16 mg of P_2_O_5_ per kg of dry soil (equivalent to 29 kg of P_2_O_5_ per ha) and compared to unfertilized treatments (control: zero P application). During the growing period, chickpea plants were fertigated with N and K_2_O at the rate of 8 and 14 mg kg^–1^ of dry soil respectively (equivalent to 15 and 25 kg ha^–1^). Ammonium nitrate (33.5% N) and potassium nitrate (13.7% N, 46% K_2_O) were used to adjust the nitrogen and potassium to the desired levels. Total quantities of the NPK fertilizers, including the Ortho-P and the Poly-P forms, were fractioned on 4 equal applications, and applied through a drip fertigation system at the rate of one application every two weeks. One dripper with a flow rate of 4 L h^−1^ was installed in each pot. The experiment was conducted in a split-plot design with six replicates per treatment.

### Stomatal density and conductance

After 55 days from sowing, nine leaflets per treatment were sampled on fully expanded leaves, located at the middle of the canopy, to assess the interactive effect of P fertilizer form and soil water content on chickpea plant gas exchange. Nine fingernail polish imprints were taken from the abaxial surface of the leaflets for each treatment, then the imprints were observed under an optical microscope (BB.1153-PLi, Euromex, Netherlands) equipped with a digital camera (CMEX-18PRO, Euromex, Netherlands). Three images were randomly selected from each imprint (27 images per treatment) and the number of stomata was counted. Using the image analysis software (Image Focus Alpha, 1.3.1.4, Euromex, Netherlands), the stomatal density was calculated by dividing the number of stomata by the area of each image. The stomatal conductance was also measured on six young and fully developed leaves per treatment using SC-1 leaf porometer (Decagon Devices, Inc. USA) after 56 days from sowing.

### Chlorophyll content index and chlorophyll a fluorescence measurement

To assess the impact of P fertilizer form on the photosynthesis apparatus of chickpea plants grown under different soil moisture conditions, the chlorophyll content index (CCI) was measured on mature and fully expanded leaves after 56 days from plant sowing. The CCI measurements were taken by portable chlorophyll meter (SPAD 502, Spectrum Technologies; Inc. USA), considering nine CCI leaf measurements per treatment. Further, the Chlorophyll *a* fluorescence (ChlF) was also evaluated to assess the impact of P fertilizer form and irrigation regime on the photosynthetic efficiency and the electron transfer into the photosystem II (PSII). Eighteen ChlF *a* measurements per treatment were performed on the youngest fully expanded leaves after 20 min of dark adaption (57 days after sowing). The measurements were taken by a portable Handy PEA fluorimeter (Plant Efficiency Analyzer, Hansatech Instruments Ltd., King’s Lynn, UK), with 3000 µmol photons m^−2^ s^−1^ of light intensity provided by an array of six light-emitting diodes (peak 650 nm) and focused on the sample surface (4 mm^2^). For each studied treatment, the fluorescence transient (OJIP curve) was recorded during the first second of leaf illumination and two photosynthetic parameters were calculated: the photosynthetic performance index (PI_ABS_) which give quantitative information about the energy conservation from photons absorbed by PSII to the reduction of intersystem electron acceptors^36^, and the total driving force DF_ABS_ as suggested to estimate the partial driving force of photosynthetic processes evaluated by the corresponding PI_ABS_^37^.

### Growth measurements

The interactive effect of the irrigation regime and P fertilizer form on chickpea growth was assessed by the measurements of two variables: specific leaf area and total leaf number per plant. The specific leaf area was measured 54 days after plant sowing on nine mature and fully expanded leaves per treatment, using a portable leaf-area meter (AM350, Netherlands), while the total leaf number per plant was taken at the harvest stage. Chickpea plants were harvested after 62 days from sowing, then shoot and root parts were carefully separated and washed with deionized water. The dried biomass was taken after 72 h of oven drying at 60 °C and the root/shoot ratio was calculated to assess the impact of different fertigation regimes on assimilates partitioning in chickpea plants.

### Chemical analyses

The dried biomass of shoot and root were separately grinded and subjected to acid digestion with 4 M HNO_3_ for the determination of macro and micronutrients content (P, K, Ca, Mg, Fe, Zn, Cu, Mn) using the ICP-AES (Agilent 5110 Inductively Coupled Plasma Optical Emission Spectrometry, USA). The total N content in shoot and root samples was measured by Kjeldahl method, in K-375 auto-analyzer (KjelMaster, Netherlands) according to O’Dell^38^. Total macro and micronutrient uptake by the plant was calculated for each treatment as the sum of the product of root and shoot dry biomass with the corresponding nutrient concentrations.

### Phosphorus and water use efficiencies

To evaluate how P fertilizer form and soil water content influence P acquisition and use by chickpea plants, P use efficiency (PUE) was calculated according to the following equation:

$$\text{PUE }(\text{\%})= \frac{\left({\text{P uptake}}_{\text{fertilized treatment}}-{\text{P uptake}}_{\text{unfertilized treatment }}\right)}{\text{amount of P supplied}}\times 100.$$

The impact of P fertilizer supply, drought stress and their interaction on the irrigation water productivity was calculated by dividing the produced shoot dry weight of each treatment on the total irrigation water used for the corresponding irrigation regime^39^.

### Statistical analysis

The interactive effects of the irrigation regime and P fertilizer form on the studied variables were analyzed through factorial design (two-way ANOVA) in SPSS data processing software (SPSS 20.0), and mean differences between treatments were evaluated by Duncan’s new multiple range test at 0.05 probability level^40^.

### Statements

All the methods were carried out in accordance with relevant guidelines and regulations. Furthermore, regulations and all experimental protocols were approved by a named institutional and/or licensing committee.

## Results

### Stomatal density and conductance

**Figure 1:**
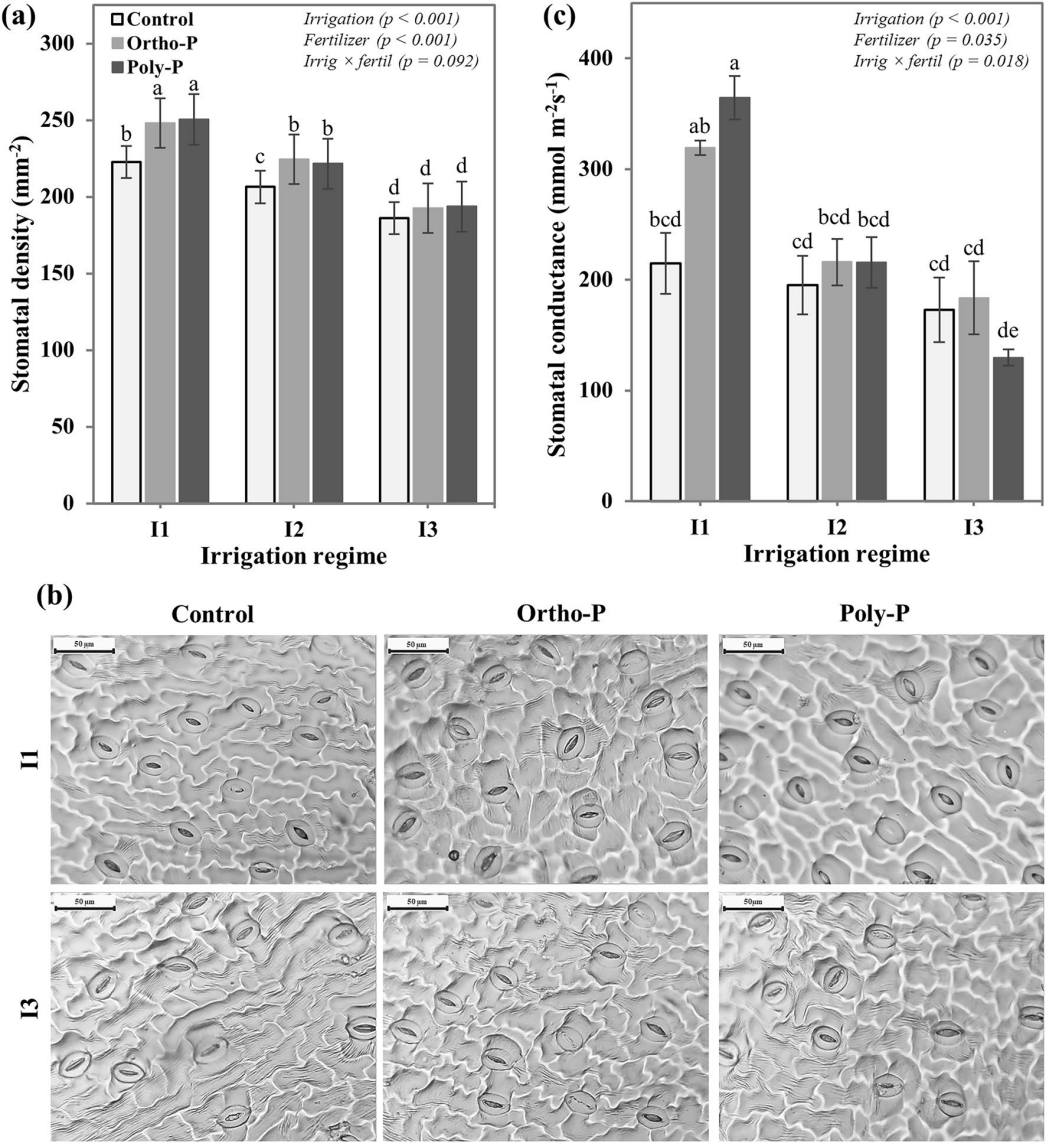
Interactive effects of P fertilizer form and irrigation regime on (**a**) leaf stomatal density and (**b**) stomatal conductance of chickpea (*Cicer arietinum*), (**c**) images of fingernail polish impressions from the abaxial leaf surface of fully expanded leaves, located at the middle of the canopy. Values are means of 6 replicates ± SE, dissimilar letters indicate significant differences at *p* < 0.05, according to Duncan’s new multiple range test.

To regulate the gas exchange with its environment, the chickpea plant significantly changed its stomatal density and conductance depending on soil moisture conditions and P nutrition regime. The obtained results in Fig. 1a revealed that the chickpea plant significantly decreased the number of stomata under I2 (50% FC) and I3 (25% FC) irrigation regimes to minimize water loss by transpiration under water stress conditions. Furthermore, the chickpea plant responded positively to P supply under adequate (I1: 75% FC) and medium soil moisture conditions (I2), increasing the stomatal density by about 12% and 7% respectively, and no significant effect was observed between P fertilizer forms. However, under severe water stress (I3), the stomatal density of chickpea leaves was not affected by P supply or P fertilizer form. Figure 1b shows stomatal images of chickpea plants grown under different drip fertigation regimes, revealing some changes in the stomata number, size, form, and opening degree, depending on P and water supply conditions. In addition to these changes in the stomatal density and morphology, P fertilizer form and irrigation regime were differently affected the stomatal conductance (Fig. 1c). The combination of Poly-P with adequate water supply (I1) resulted in higher stomatal conductance with a 70% increase as compared to the control treatment, followed by the Ortho-P with 45%. The amplitude of the positive effect of P fertilizer on stomatal conductance was significantly decreased with water stress regimes. Although both P fertilizers (Orth-P and Poly-P) increased the stomatal conductance by about 8% compared to the unfertilized treatment, the mean comparison did not reveal any statistical differences. Moreover, under severe water stress (I3), the Ortho-P application did not affect the stomatal conductance, and plants fertigated with Poly-P recorded a lower stomatal conductance than the unfertilized plants.

### Chlorophyll content and photosynthetic efficiency

**Figure 2:**
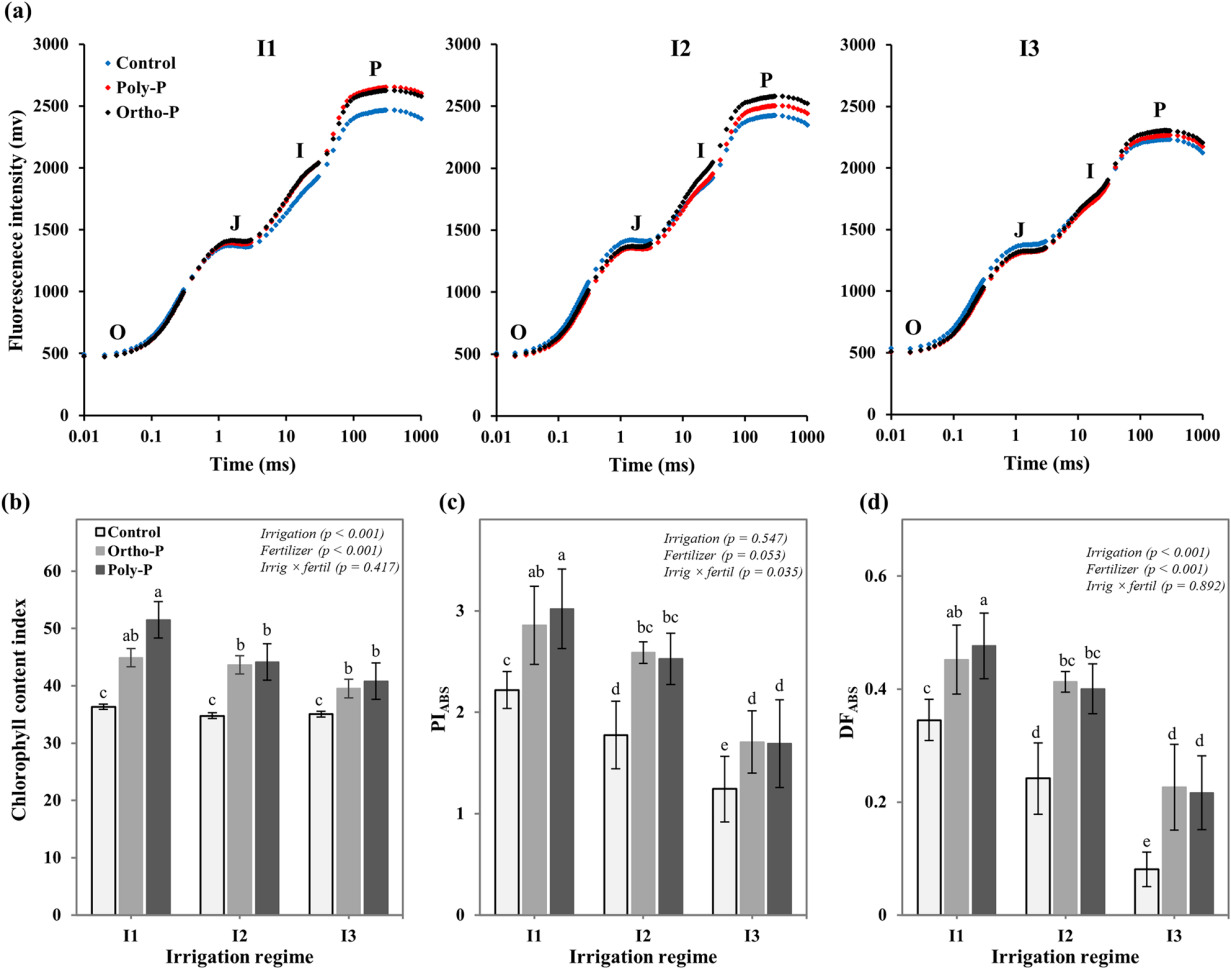
Interactive effects of P fertilizer form and irrigation regime on (**a**) Chl *a* fluorescence OJIP transient curves, (**b**) chlorophyll content index (*Cicer arietinum*), (**c**) photosynthetic performance index (PI_ABS_) for energy conservation from photons absorbed by PSII to the reduction of intersystem electron acceptors, and (**d**) driving force on absorption basis, suggested for estimating the driving force of processes evaluated by the corresponding PI_ABS_ (DF_ABS_) in chickpea plants (*Cicer arietinum*). Values are means of 6 replicates ± SE, dissimilar letters indicate significant differences at *p* < 0.05, according to Duncan’s new multiple range test.

Chlorophyll content, as assessed by SPAD measurements on fully expanded leaves, was significantly increased with P supply for all studied irrigation regimes (Fig. 2b). Under adequate soil moisture conditions (I1), the Poly-P and the Ortho-P fertilizers improved the chlorophyll content index (CCI), by 42% and 23% respectively, compared to the unfertilized treatment. However, under medium and severe water stress conditions (I2 and I3), both P fertilizers increased the CCI by about 25% and 14% respectively, and no significant differences were observed between P forms.

Data in Fig. 2a revealed that all P and water regimes showed a typical shape of the chlorophyll fluorescence curves with the polyphasic O-J-I-P steeps. The initial fluorescence intensity F_O,_ which refers to minimal fluorescence when RCs are open, was slightly increased under drought stress regimes (I2 and I3) comparatively to that under well-watered conditions (I1), while the maximum fluorescence F_m_, referring to maximal fluorescence when RCs are closed, was significantly decreased. A positive effect was also observed on the F_m_ in response to different P fertilizer applications, especially under adequate irrigation and medium water stress regimes. The changes in the O-J-I-P transients were more pronounced at the K-steep (300 µs) and the I-P phase, particularly under moderate drought conditions and P fertigated treatments.

These changes in the fluorescence curves following the application of the different P fertilizer forms, resulted in an improvement of the photosynthetic performance index PI_ABS_, in all studied irrigation regimes, with pronounced values under full irrigation (I1) and medium water stress conditions (I2). Regarding the mean values per irrigation regime, we observed a significant decrease in the PI_ABS_ of plants exposed to medium and severe water stress (Fig. 2c). Similar trends were also observed for the driving force parameter (DF_ABS_), in response to the different combinations of water and P fertilizer regimes (Fig. 2d).

### Specific leaf area and total leaf number

**Figure 3:**
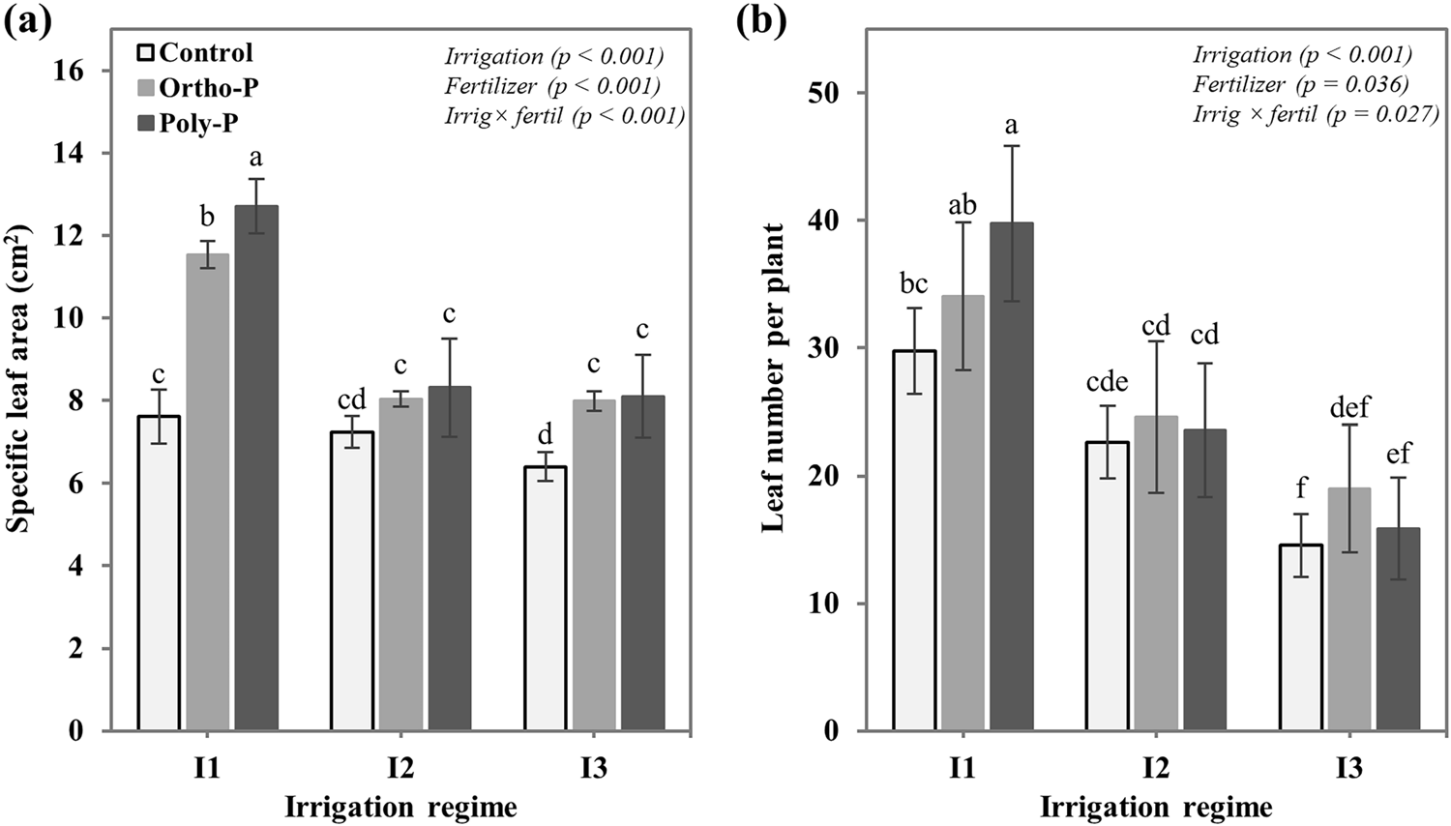
Interactive effects of P fertilizer form and irrigation regime on (**a**) specific leaf area and (**b**) total leaf number per plant in chickpea (*Cicer arietinum*). Values are means of 6 replicates ± SE, dissimilar letters indicate significant differences at *p* < 0.05, according to Duncan’s new multiple range test.

Chickpea plants responded differently to P application and P fertilizer form under different soil moisture conditions. Results in Fig. 3a showed that P supply significantly increased plant growth, as assessed by the measurement of the specific leaf area and total leaf number per plant, with significant values under I1 irrigation regime. Under adequate soil moisture conditions (I1: 75%FC), the specific leaf area of plants fertigated with the Ortho-P and Poly-P forms was respectively increased by 51% and 67%, as compared to the unfertilized plants. Nevertheless, this parameter was significantly decreased under water limitation conditions (I2 and I3) for all studied P nutrition regimes. Similarly, for the total leaf number per plant, the obtained results revealed a significant impact of the interactive effect of P fertilizer form and the irrigation regime. Data in Fig. 3b demonstrates that P application resulted in a higher leaf number per plant. However, some interactive effects of P form and irrigation were observed. The Poly-P fertilizer gives significantly higher values of total leaf number per plant under adequate and medium irrigation regimes (I1: 40 leaves and I2: 24 leaves). While under severe water stress conditions, the effectiveness of Poly-P was considerably reduced (I3: 16 leaves), contrary to the Ortho-P form which keep its positive effect on leaf development ever under water stress conditions, with a 30% of increase as compared to the unfertilized plants.

### Biomass accumulation and partitioning

**Figure 4:**
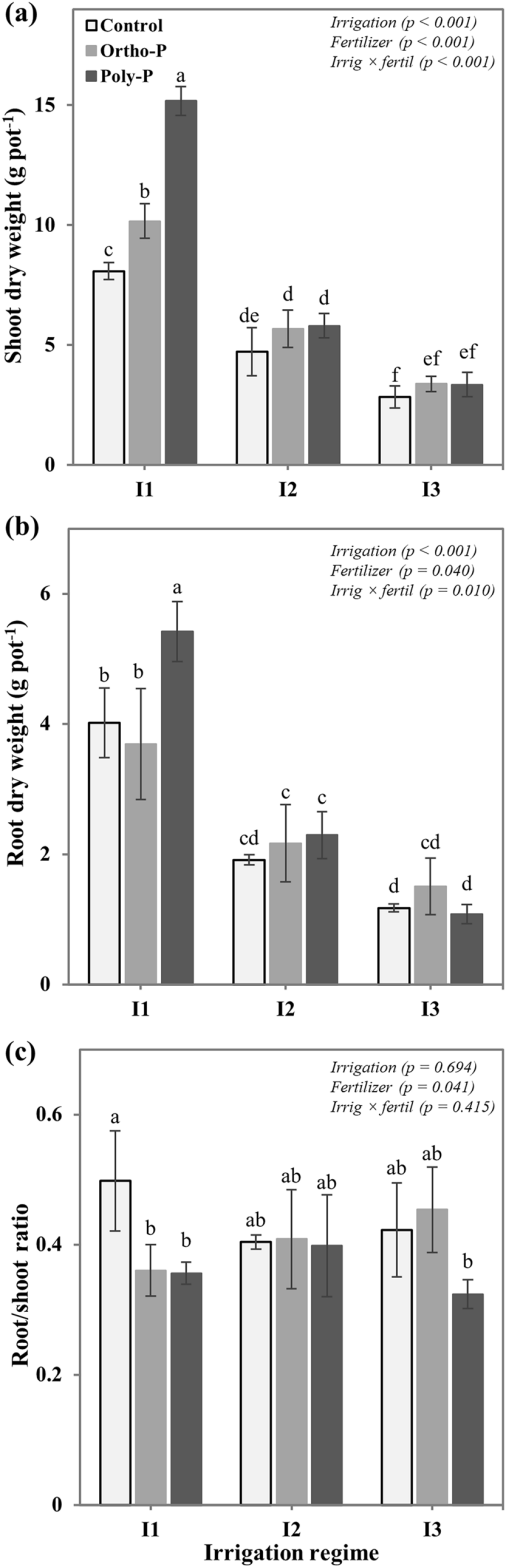
Interactive effects of P fertilizer form and irrigation regime on (**a**) shoot dry weight, (**b**) root dry weight, and (**c**) root/shoot ratio of chickpea (*Cicer arietinum*). Values are means of 6 replicates ± SE, dissimilar letters indicate significant differences at *p* < 0.05, according to Duncan’s new multiple range test.

Plant biomass accumulation was greatly improved in response to P and water supply. The data in Fig. 4 show that under full irrigation regime (I1), plants fertigated with Poly-P fertilizer accumulated 1.88 times more shoot dry weight (SDW) than the control treatment, followed by the Ortho-P which increased SDW by 25% compared with the unfertilized treatment. A significant interaction between P fertilizer form and irrigation regime was observed. Results in Fig. 4a shows that a reduction in soil water content (I2 and I3) resulted in significant decreases of SDW for all studied treatment. Even though both P fertilizers (Poly-P and Ortho-P) increased the chickpea shoot biomass by about 21% and 18% under I2 and I3 irrigation regimes, respectively over the unfertilized treatment, the mean comparison did not reveal any statistical differences between the fertilized treatments and the control. Regarding root dry weight (RDW), the obtained results revealed that P fertilizer form, irrigation regime, and their interactions significantly influenced root growth in chickpea (Fig. 4b). Comparing the mean values of each irrigation regime, we observed that chickpea root growth was significantly decreased under medium and severe water stress (I2 and I3), compared to an adequate water supply regime (I1). The Poly-P applied with full irrigation recorded the highest value of RDW, 1.35 times more than the control. The intensity of this improvement of root growth with Poly-P was significantly reduced under water stress regimes.

To evaluate how P fertilizer form and irrigation regime affect the carbohydrate allocation in chickpea plants, the root/shoot ratio was calculated. Results in Fig. 4c show that the unfertilized plant recorded the highest root/shoot ratio of 0.49, which means that plants allocate much more photosynthesis assimilates to the development of the belowground part. However, under medium water stress conditions (I2), no significant effect was observed between the studied P nutrition regimes, and a slight decrease of root/shoot ratio was noticed in plants fertigated with Poly-P under severe water stress conditions (I3).

### Phosphorus uptake and P and water use efficiency

**Figure 5:**
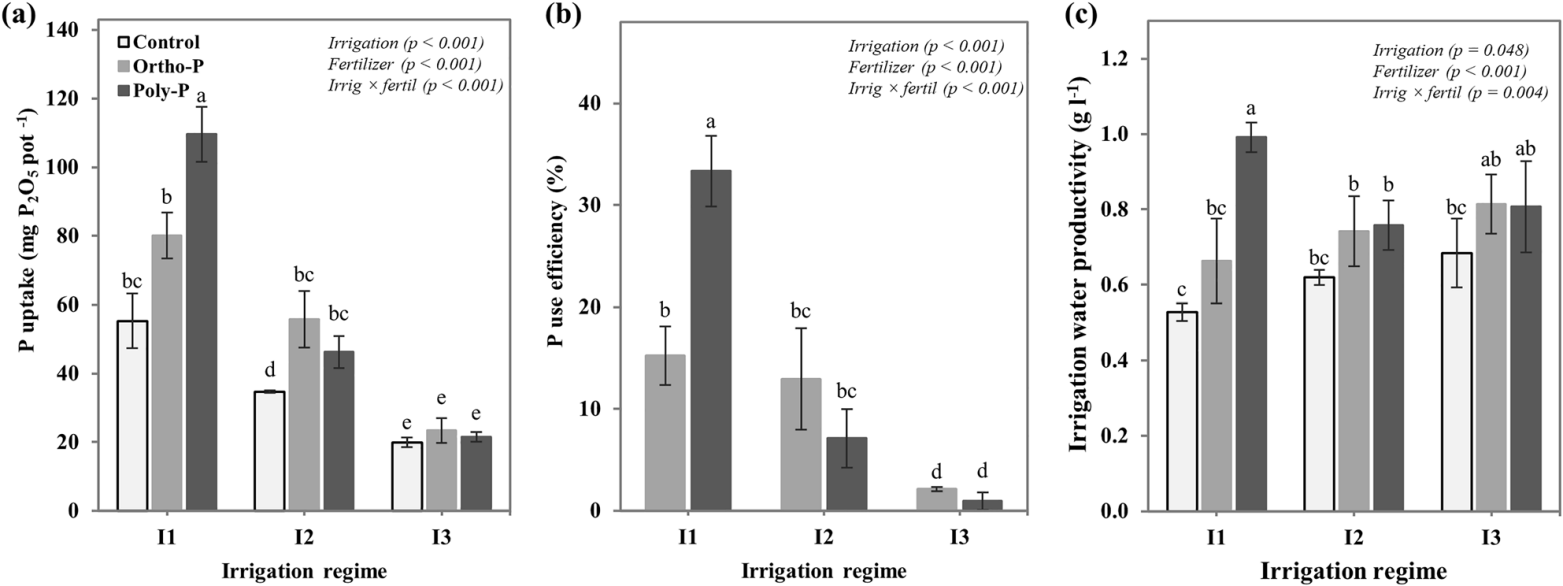
Interactive effects of P fertilizer form and irrigation regime on (**a**) phosphorus uptake, (**b**) phosphorus use efficiency, and (**c**) irrigation water productivity of chickpea (Ci*cer arietinum*). Values are means of 6 replicates ± SE, dissimilar letters indicate significant differences at *p* < 0.05, according to Duncan’s new multiple range test.

Phosphorus uptake by chickpea plants was significantly influenced by the interactive effect of P fertilizer form and irrigation regime. As presented in Fig. 5a, P application significantly improved P uptake, with increasing values under non-limited water supply conditions (I1). Chickpea plants grown under Poly-P and I1 fertigation regimes recorded the highest value of P uptake, 1.98 times higher than that of the unfertilized treatment, followed by the Ortho-P which accumulated 20% of P over the control. Additionally, under medium water stress (I2), the Ortho-P application resulted in higher P accumulation by chickpea plants, with 60% of P uptake over the control, contrary to the Poly-P which recorded 33%. However, no significant effect of P application or P fertilizer form was observed under severe water stress (I3). Data in Fig. 5b showed the effect of the studied treatments on P use efficiency (PUE). Regarding these results, we observed that under non-limiting water supply (I1), Poly-P fertilizer form recorded 33% of PUE, contrary to the Ortho-P form which recorded 16%. However, under water stress conditions, PUE was significantly reduced, mainly for the Poly-P form which recorded 7% and 2% of PUE under I2 and I3, respectively.

Regarding the impact of the studied irrigation regimes on the irrigation water productivity (IWP), it can be seen in Fig. 5c that the IWP was significantly increased under moderate (I2) and severe water stress (I3), compared to the full irrigation regime (I1), especially for plants fertigated with the Ortho-P form and the unfertilized treatment. Compared to the well-watered regime, the Ortho-P fertilizer increased the IWP by 12% and 23% in I2 and I3 regimes, respectively, while the unfertilized treatment increased the IWP by 17% and 30%. Nevertheless, the Poly-P fertilizer application resulted in a higher value of IWP (0.99 g L^−1^) when applied under well-watered conditions (I1), although under moderate and severe water stress regimes the Poly-P fertilizer recorded values like those of the Ortho-P.

### Nutrient uptake

Soil water content is one of the main factors influencing plant nutrient uptake, and it is widely known for its primordial role in solutes transport to roots, nutrient solution equilibrium, and microbial activity improvement. The obtained results revealed that chickpea plants responded differently to P supply, P fertilizer form, and their interactions with the irrigation regimes. Data in Table 1 indicate that chickpea plants grown under a full irrigation regime (I1) absorbed much more macro and micronutrients than those grown under medium and severe water stress, with significantly higher values under the Poly-P fertigation regime. As compared to the unfertilized treatment, the combination of Poly-P with adequate irrigation level (I1) increased macro (N, K, Ca, and Mg) and micronutrients (Fe, Zn, Cu, and Mn) uptake by about 41% to 72%, depending on the nutrient. Also, under the Ortho-P fertigation regime, macronutrient absorption was improved by about 7% to 36% over the control. In contrast to the Poly-P form which significantly improved micronutrient uptake, a slight decrease in Zn and Cu uptake was noticed for the Ortho-P form. These trends in nutrient uptake were not conserved under medium and severe water stress, and significant interactive effects of P fertilizer form and irrigation regime were observed. Under the I2 irrigation regimes, P application resulted in a slight improvement of N, K, Mg, Fe, Cu, and Mn uptake compared to the control, and no significant differences were observed between P fertilizer forms for Mg and Mn uptake. However, under severe water stress conditions, significant differences were observed between P fertilizer forms. The Ortho-P application resulted in higher nutrient uptake than Poly-P, which accumulated less amount of Mn, Zn, and Cu compared to the unfertilized treatment.

**Table 1:** Interactive effect of irrigation regime and P fertilizer form on chickpea macro and micronutrient uptake.

Irrigation regime	Fertilizer	Nutrient uptake (mg pot^−1^)							
		N	K	Ca	Mg	Fe	Zn	Cu	Mn
I1	Control	199.1 ± 10^c^	332.3 ± 21^b^	219.4 ± 31^b^	81.7 ± 13^b^	4.3 ± 0.7^b^	0.36 ± 0.06^b^	0.103 ± 0.02^b^	0.414 ± 0.08^b^
	Ortho-P	271.4 ± 17^b^	363.3 ± 69^b^	238.2 ± 39^b^	87.8 ± 21^b^	6.5 ± 1.5^a^	0.28 ± 0.05^bc^	0.087 ± 0.02^bc^	0.494 ± 0.14^b^
	Poly-P	315.0 ± 8^a^	555.2 ± 60^a^	341.4 ± 13^a^	128.1 ± 13^a^	7.0 ± 1.2^a^	0.62 ± 0.19^a^	0.146 ± 0.04^a^	0.693 ± 0.03^a^
I2	Control	158.1 ± 16^d^	177.6 ± 5^cd^	117.9 ± 1^c^	36.7 ± 1^cd^	2.8 ± 0.2^cd^	0.16 ± 0.01^cd^	0.051 ± 0.01^de^	0.195 ± 0.01^cd^
	Ortho-P	169.7 ± 24^d^	216.8 ± 49^c^	143.1 ± 33^c^	46.9 ± 9^c^	2.5 ± 0.7^cd^	0.18 ± 0.03^cd^	0.056 ± 0.01^d^	0.255 ± 0.06^c^
	Poly-P	176.8 ± 12^cd^	200.7 ± 29^cd^	138.8 ± 16^c^	45.0 ± 5^c^	3.4 ± 0.7^bc^	0.19 ± 0.03^cd^	0.060 ± 0.01^cd^	0.247 ± 0.05^c^
I3	Control	86.9 ± 11^e^	104.2 ± 9^e^	50.2 ± 9^d^	19.8 ± 2^d^	1.76 ± 0.3^d^	0.11 ± 0.01^d^	0.030 ± 0.01^de^	0.119 ± 0.02^d^
	Ortho-P	103.8 ± 6^e^	139.0 ± 25^de^	67.2 ± 14^d^	29.4 ± 11^cd^	2.4 ± 0.7^cd^	0.13 ± 0.02^d^	0.036 ± 0.01^de^	0.175 ± 0.05^cd^
	Poly-P	98.2 ± 10^e^	103.2 ± 7^e^	54.6 ± 8^d^	19.4 ± 2^d^	1.8 ± 0.3^d^	0.09 ± 0.01^d^	0.023 ± 0.01^e^	0.117 ± 0.02^d^
***p* value**									
Irrigation		< 0.001	< 0.001	< 0.001	< 0.001	< 0.001	< 0.001	< 0.001	< 0.001
Fertilizer		< 0.001	0.001	0.001	0.009	0.019	0.020	0.092	0.007
Irrig × Fertil		< 0.001	< 0.001	0.001	0.004	0.037	0.002	0.017	0.008

Data are mean values ± SE (n = 6), dissimilar letters indicate significant differences at *p* < 0.05, according to Duncan’s new multiple range test.

## Discussion

Under nutrient and water stress conditions, plants manifest multiple morphological and physiological changes to improve their capacity to absorb and use water and mineral resources^41–43^. It is well documented that the individual or the combined effects of drought and P deficiency affect photosynthetic activity, nutrient uptake, and plant growth and yield^35,42,44,45^. The previous studies dealing with the interaction between water and P were mainly focused on the effect of P rate applied or P concentration in the soil solution on crop growth and development^20,34,46^. To the best of our knowledge, this is the first study that investigated the interactive effects between P fertilizer form (with a focus on Ortho-P and Poly-P) and soil water content on chickpea photosynthetic activity, plant growth, water and nutrient uptake, and their use efficiency. The response of the chickpea plants to Poly-P and Ortho-P fertilizers was studied by the simulation of three probable scenarios of soil water content (adequate water supply, medium, and severe water stress) in the Mediterranean region, strongly characterized by longer and very frequent drought episodes.

The present study showed significant effects of P fertilizer form and irrigation regime as well as their interaction on the chickpea leaves. The application of P fertilizers significantly increased the stomata density under well-watered (I1) and medium drought regimes (I2) compared to the unfertilized treatment (Fig. 1a). However, under severe water stress conditions, the positive impact of P supply on stomatal density was drastically reduced. The same trends were observed for the stomatal conductance which reflects the level of gas exchange between chickpea leaves and their environment. Phosphorus and water supply resulted in a significant increase of the stomatal conductance, and Poly-P recorded the higher value under well-watered conditions (Fig. 1c). These results suggest that the P fertilizer form which was able to provide chickpea plants with sufficient quantity of available P recorded higher stomatal density and conductance compared to the unfertilized control^46^, hence, the combination of Poly-P and full irrigation regime (I1) resulted in the highest stomata density and conductance. Contrary to the Poly-P which greatly reduced the stomatal density under drought stress conditions (I2 and I3), the Ortho-P fertilizer kept its positive effect on stomatal density and conductance even under water stress regimes. These differences between P fertilizer forms can be explained by the impact of soil water content on the availability of P in soil. Under drought conditions, available P from the Ortho-P fertilizer is expected to be higher than that of the Poly-P since the hydrolysis of Poly-P is greatly reduced under water stress conditions^47,48^. Similar results were found by Sekiya and Yano^46^, who reported that an increase in superphosphate (which are Ortho-P fertilizer) application rate improved cowpea stomatal density under different water supply conditions. The authors revealed also that the improvement of the stomatal density with P supply was more pronounced under elevated atmospheric CO_2_ concentrations. According to Sharma et al.^49^, chickpea plants grown under drought and low P supply conditions reduced significantly their stomatal conductance to avoid leaf turgor loss via the transpiration process.

**Figure 6:**
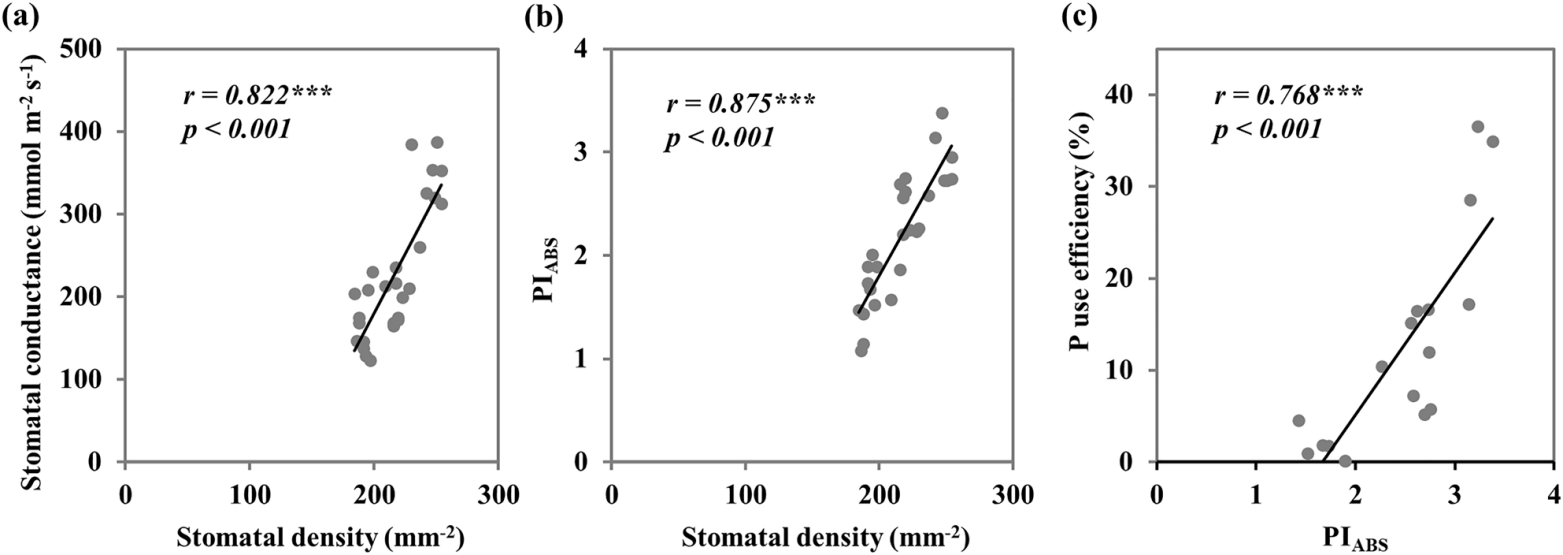
Correlations between stomatal density and (**a**) stomatal conductance, (**b**) photosynthetic performance index (PI_ABS_), and (**c**) between PI_ABS_ and phosphorus use efficiency in chickpea plants (*Cicer arietinum*) grown under different P fertilizer forms and irrigation regimes. Asterisks *, **, and *** denote Pearson correlation significance at *p* < 0.05, *p* < 0.01, and *p* < 0.001, respectively.

Chlorophyll content of chickpea leaves was significantly increased with P supply under all irrigation regimes, with no significant difference between P fertilizer forms. However, a remarkable decrease of chlorophyll content was observed under drought stress conditions (Fig. 2b). These results confirm our previous finding on the impact of the Ortho-P and Poly-P forms on chickpea chlorophyll content^25^. Similar results were also found by Liu et al.^50^ and Pingoliya et al.^51^, who reported that water stress and P deficiency resulted in a decrease of the leaf chlorophyll content since these abiotic stress increased chlorophyll pigment degradation. These variations in the chlorophyll content as well as in the stomatal density and conductance have strongly impacted the photosynthetic activity of chickpea plants. As shown in Fig. 2a, P and water supply had significant effects on the linear electron flow between the PSII and PSI, which had highly influenced chickpea photosynthetic performances (Fig. 2c,d). Plants grown under Poly-P and full irrigation regime (I1) reached the maximum fluorescence (Fm) and recorded the highest values of the photosynthetic performance index PI_ABS_ and driving force parameter DF_ABS_. However, under medium and severe water stress, the capacity of the Poly-P to enhance chickpea photosynthesis was greatly reduced, and the Ortho-P form recorded the highest values of PI_ABS_ and DF_ABS_. Close relationships were established between leaf stomatal density, stomatal conductance (*r* = 0.82), and photosynthetic performance index PI_ABS_ (*r* = 0.87) (Fig. 6a,b). These correlations revealed the importance of stomata density, size, and opening degree in carbon fixation and photosynthesis efficiency^52–54^. The individual effect of P supply on the photosynthetic activity of chickpea plants has been extensively explained by Carstensen et al.^55,56^ and Chtouki et al.^25^, showing a significant improvement of the electron transport rate with P application, especially in the thermal phase J-I-P. Regarding the obtained results in the present study, we suggest that the interactive effects of P and soil water content on the photosynthetic activity may be explained by the aptitude of the studied P fertigation regimes (P form and water regime) to supply chickpea plants with sufficient amount of P and water and also by their indirect effects on the uptake of other nutrients, mainly those directly involved in the electron transport^57–59^. As shown in Table 1, chickpea plants fertigated with Poly-P under well-watered conditions absorbed much more quantities of micronutrient (Fe, Cu, Mn) compared to the Ortho-P and the unfertilized treatments. These findings can be explained by the positive effect of Poly-P fertilizer on micronutrients availability in the soil as revealed by Gao et al.^24^, who reported that Poly-P fertilizer had the capacity to chelate micronutrients (Fe, Zn, and Mn), which significantly improved their uptake in maize plants under alkaline soil conditions. However, in our study, we demonstrate that strong chelation of micronutrients by the Poly-P may greatly reduce their availability for plants, especially when the hydrolysis process of the Poly-P fertilizer is impacted due to the low soil moisture content (Table 1).

Our findings related to chickpea growth attributes revealed that an adequate P and water supply resulted in higher leaf specific area, higher leaf number per plant, and greater shoot dry matter. The combination of the Poly-P form with the I1 irrigation regime recorded the best results in terms of canopy growth and biomass accumulation. Significant interactive effects between P fertilizer form and soil water content were observed. Regarding results in Figs. 3 and 4, we find that the response of chickpea crop to the Poly-P application is strongly influenced by soil moisture content. Although the Poly-P fertilizers have beneficial effects on plant photosynthesis and growth under optimal irrigation conditions^25,26,60–62^, their effectiveness is greatly reduced under water stress conditions, unlike the Ortho-P fertilizer which exhibits a stable effect on plant growth even under water stress conditions. These changes in chickpea plants’ phenotype (specific leaf area and canopy) and growth (biomass accumulation and allocation) can be associated with the impact of different water and P supply regimes on photosynthesis efficiency^63–65^. Plants exposed to prolonged drought stress significantly reduced their stomatal density and conductance which greatly decreased the photosynthesis activity as assessed by the PI_ABS_ index and consequently reduced the CO_2_ fixation and biomass accumulation^46^. Moreover, the allocation of the photosynthetic assimilates may be changed. As revealed in Fig. 4b, plants exposed to medium drought or P deficiency increased the root/shoot ratio to improve their capacity to absorb water and nutrient^66–68^.

In this regard, we hypothesized that changes in soil moisture content and P availability in soil drive the stomata functioning (Fig. 1) and photosynthesis activity (Fig. 2), which in return impact nutrient uptake and use efficiency and consequently biomass accumulation and allocation. The correlation established between the photosynthesis efficiency (PI_ABS_) and P use efficiency supported our hypothesis (Fig. 6c). The obtained results in this study revealed that P uptake and use efficiency increased significantly with P fertilizer application and decreased under water stress. Following the same trends in photosynthesis efficiency and biomass accumulation, the Poly-P fertilizer reached the maximum P uptake and use efficiency when applied in well-watered conditions (I1), however, under water stress conditions its efficiency in terms of nutrients uptake was significantly reduced. The increased P uptake in the Poly-P-I1 treatment was accompanied by significant increases in nutrient uptake (N, K, Fe, Zn, Cu, and Mn). These results may be explained by the positive synergy between P, N and K and by the aptitude of Poly-P to chelate and release micronutrients for plant roots^24,69,70^. However, for the Ortho-P form, our results revealed that chickpea plants kept a stable response in terms of P uptake improvement under all studied water irrigation regimes. Moreover, the application of P fertilizers significantly improved water productivity in chickpea plants, especially under water stress conditions. These results confirmed the importance of adequate P nutrition in the mitigation of drought stress effects on plants growth and productivity^19,35,49^.

## Conclusions

The results suggested that the stomatal density and conductance, which play an important role in the adjustment of plants’ gas exchanges with the environment under drought and nutrient-deficient conditions, induce a cascade of actions linked to photosynthesis efficiency, nutrients uptake, and biomass accumulation and allocation. The interactive effect between phosphorus nutrition and water supply regimes indicated that P fertilizer form has a significant influence on chickpea biophysiological processes. Although both P fertilizer forms (orthophosphates and polyphosphates) significantly improved stomatal conductance, photosynthetic activity, biomass accumulation, and nutrient uptake, their effectiveness is strongly influenced by soil water content. The obtained results clearly showed the significant effect of polyphosphate fertilizer forms on chickpea response to P supply (greater plant growth and P use efficiency). Therefore, water availability remains an important point to be considered for any eventual integration of the Poly-P fertilizer forms in the crop fertilization programs under Mediterranean conditions, especially for annual crops, highly dependent on P nutrition at their early growth stages.

## Data Availability

All data generated or analyzed during this study are included in this article. Excel files can be provided on demand and should be addressed to A.O.
